# Isolation and characterization of high exopolysaccharide-producing *Weissella confusa* VP30 from young children’s feces

**DOI:** 10.1186/s12934-019-1158-1

**Published:** 2019-06-13

**Authors:** Hui Jin, Yunju Jeong, Sang-Ho Yoo, Tony V. Johnston, Seockmo Ku, Geun Eog Ji

**Affiliations:** 1 Department of Food and Nutrition, Research Institute of Human Ecology, Seoul National University, Seoul, 08826 South Korea; 2 Research Center, BIFIDO Co., Ltd., Hongcheon, 25117 South Korea; 3 Department of Food Science & Biotechnology, and Carbohydrate Bioproduct Research Center, Sejong University, Seoul, 143-747 South Korea; 4 Fermentation Science Program, School of Agriculture, College of Basic and Applied Sciences, Middle Tennessee State University, Murfreesboro, TN 37132 USA

**Keywords:** Exopolysaccharide, *Weissella confusa*, Dextran, Lactic acid bacteria, Safety, Structure

## Abstract

**Background:**

Lactic acid bacteria (LAB) are known to have a significant ability to colonize the human intestinal tract and adhere to the surface of intestinal epithelial cells. Among the various lactic acid bacteria, exopolysaccharide (EPS) producing strains are known to provide a variety of health benefits for their hosts (e.g. anti-inflammatory, antioxidant, antitumor and stress tolerant effects). Recently, EPSs and EPS-producing lactic acid cultures have gained interest within the food industry and are playing important roles as biothickeners and texturizing agents due to their hydrocolloidal nature. In this study, 156 lactic acid bacterial strains isolated from fecal samples of healthy young children were screened and evaluated for active EPS-production capability.

**Results:**

Among the various human origin lactic acid flora isolated, *Weissella confusa* VP30 showed the highest EPS productivity and its EPS producing properties were characterized under various cultural conditions in this research. To document the safety of *W. confusa* VP30, antibiotic resistance, hemolytic, and ammonia production properties were evaluated in addition. No significant negative results were observed. The maximum EPS production by *W. confusa* VP30 was 59.99 ± 0.91 g/l after 48 h of cultivation in media containing 10% sucrose, far exceeding EPS production by other bacterial strains reported elsewhere. Based on gel permeation chromatography (GPC), the molecular weight of EPS produced by *W. confusa* VP30 was 3.8 × 10^6^ Da. Structural analysis of the released EPS fraction by ^13^C and ^1^H nuclear magnetic resonance (NMR) spectroscopy revealed that *W. confusa* VP30 can produce dextran with glucose units linked with 96.5% *α* (1 → 6) glycosidic bonds and 3.5% *α* (1 → 3) branches.

**Conclusion:**

The high EPS production capability and safety of *W. confusa* VP30 justify food industry consideration of this cell strain for further evaluation and potential industrial use.

## Introduction

Numerous LAB genera, including *Carnobacterium*, *Enterococcus*, *Lactobacillus*, *Lactococcus*, *Leuconostoc*, *Oenococcus*, *Pediococcus*, *Streptococcus*, *Tetragenococcus*, *Vagococcus* and *Weissella* sp. have been isolated from a variety of human, animal, plant and environmental sources and widely utilized for processed food production [1–4]. According to recent industry analysis reports [5, 6], the global LAB and probiotics market is characterized by three applications and/or end-uses: (i) probiotics in foods and beverages [e.g. cereal, dry, dairy, non-dairy, baked food and fermented meat products], (ii) probiotic dietary supplements [e.g. nutraceuticals, single cell protein, specialty nutrients, infant formula], and (iii) animal feed probiotics [e.g. substitutes of antibiotic-based growth stimulants]. According to Global Market Insights [7], the US and EU markets were estimated at over USD 1.8 billion and 630 million, respectively, in 2017. This three-part market of significant value is projected to grow even further due to increased consumer interest in health and expanding consumer income. An increasing number of food and feed companies are therefore trying to utilize LAB in their products to meet customer demand, improve product quality, and diversify their product lines [8].

Among LAB strains, human origin LAB strains have shown high resistance to gastric juice and bile as well as high survival rates in the gastrointestinal tract, due to their ability to adhere to the surface of intestinal epithelial cells [8, 9]. Multiple researchers have demonstrated the diverse biofunctionalities (e.g. anti-allergic effects, anticancer properties, bacteriocin production, high transmission rates and high adherence ability) of human origin LAB and their metabolic substances through in vitro and in vivo experiments [10–14]. Due to the verified safety and functional effects of human origin LAB, these special LAB have been widely examined and applied to foods and livestock feeds as supplements, resulting in steadily increasing market demand [5, 6]. Therefore, the isolation of novel functional human origin intestinal bacteria and characterization of their bioactive molecules are important for the production of new value-added products [8]. The screening of novel microorganisms and characterization of their metabolites is regarded as the first step in their utilization as probiotics and drugs, as well as for metabolic engineering [15–17].

Among the bioactive molecules produced by LAB, there is growing demand for EPSs, which are used as biobased polymers [18]. EPS has been widely studied for its economic importance in food processing and beneficial effects. EPS produced by LAB can act as a viscosifier and/or emulsifying agent and play an important role in the processing of yogurt, cheese, and other fermented foods as a stabilizer [19]. Multiple groups have reported that EPS from LAB has significant biofunctional properties and its functionality varies depending on structure and the bacterial strain which produced it. For example, EPS derived from *Lactobacillus rhamnosus* KL37 has been shown to suppress active arthritis by the inhibition of the production of arthritogenic antibodies [20]. According to London et al. [21], β-glucan enriched EPS from *Lactobacillus paracasei* NFBC 338 showed modulatory effects on lipid metabolism. EPS produced by *Streptococcus thermophilus* ST10 and *Weissella cibaria* GA44 exhibited physical defense functionality that strengthens the tight junction between human intestinal cells [22], and antioxidant effects [23], respectively. Despite these promising food applications and biofunctionalities, EPS is not yet widely utilized by food processors as a food additive, mainly due to low production rates by LAB [24]. Therefore, screening for novel LAB with high EPS production rates is justified.

In this work, high EPS producing LAB strains with potential food processing applications were isolated from young children’s fecal samples and characterized. One hundred fifty-six human origin LAB strains were screened. All 156 LAB samples were inoculated and cultured in seven modified de Man, Rogosa, and Sharpe (MRS) agar (Becton, Dickinson, and Company) medias, each with a different carbon source. The amount of EPS produced from each of the 1248 different groups was qualitatively and/or quantitatively analyzed. Based on our results, the highest EPS-producing LAB (*Weissella confusa* VP30) and its key carbon source (sucrose) for high EPS production were determined. Further structural characterization using NMR analysis allowed us to elucidate the conformation of EPS from *W. confusa* VP30. This strain is also safe to use as an edible LAB from a food safety standpoint, according to our previous research [25].

## Materials and methods

### Sampling and isolation of LAB from young children’s feces

Using a study protocol approved by the Seoul National University Institutional Review Board (IRB No. 1702/002-013), fresh fecal samples were collected from five young children between 1 and 6 years old for this study. The fecal samples were transported to the laboratory at 4 °C for LAB isolation in a laminar flow hood. One gram samples from each feces collected was mixed with a sterilized phosphate buffered saline solution (PBS; 137 mM NaCl, 2.7 mM KCl, 4.3 mM Na_2_HPO_4_, 1.47 mM KH_2_PO_4_, pH 7.4) [26]. After serial dilution, the suspension of each sample was plated on *Lactobacillus* Selection (LBS) (Difco) agar or transgalctosylated oligiosaccharide (TOS-MUP) (MB cell) agar and incubated under anaerobic conditions at 37 °C for 48 h for LAB isolation. LBS [27, 28] and TOS-MUP [29, 30] were used to screen *Lactobacillus* and *Bifidobacterium* spp. from fecal samples, respectively.

One hundred fifty-six morphologically different microbial colonies were randomly collected and the selected colonies were Gram stained and morphologically evaluated using an optical microscope. The isolated LAB strains were then cultured in modified MRS media according to our previous studies [9, 26, 31, 32]. In brief, all 156 LAB samples were inoculated and cultured in seven types of modified MRS agar media with different carbon sources [10% (w/v) of glucose (Sigma), sucrose (Sigma), galactose (Sigma), lactose (Difco), fructose (Sigma), raffinose (Sigma) and maltose (Sigma)] and commercially available MRS media (1248 total samples).

According to Ruas-Madiedo and De Los Reyes-Gavilán [33], mucinous strands can be observed visually when sterilized inoculation loop are contacted with microbial colonies that produce EPS as a metabolite. Therefore, we conducted an initial screening using a sterilized inoculation loop or sterilized toothpicks to screen for EPS production candidates. From the 1248 samples, the 10 most mucoid and slimy colonies and/or media conditions were selected for further study. 16S rRNA gene sequencing was performed to accurately identify these 10 strains at the molecular level.

### Phylogenetic analysis

The selected microbial isolates were then subjected to phylogenetic analysis. DNA from each isolate was isolated, amplified by PCR, and the 16S rRNA genes in the PCR products were sequenced. The PCR primers used were 27F (5′-AGAGTTTGATCMTGGCTCAG-3′)/1492R (5′-TACGGYTACCTTGTTACGACTT-3′) and the 16 s rRNA sequencing primer was 785F (5′-GGATTAGATACCCTGGTA-3′)/907R (5′-CCGTCAATTCMTTTRAGTTT-3′). The PCR sequencing step was executed by Macrogen, Inc. (Seoul, Korea), and the full sequence of 16S rRNA gene was aligned using the NCBI database, to the species level [34].

### Analysis of EPS production properties of 10 LAB strains

The level of EPS production of 10 LAB strains cultivated in modified MRS broth containing 10% (w/v) sucrose instead of glucose was enumerated and compared using our previously published protocol with slight modifications [9, 26, 31, 32]. Specifically, cultured bacteria was boiled for 15 min and treated with 17% (v/v) 85% trichloroacetic acid (TCA) (Sigma) 2 h after cell cultivation for 24 h at 37 °C. LAB pellets were separated from culture media via centrifugation at 18,000×*g* for 25 min [35]. A 5 ml aliquot of the supernatant was mixed with 25 ml of 95% ethanol (− 20 °C), and incubated at 4 °C for 16 h. The precipitated EPS was centrifuged at 18,000×*g* for 20 min, and the supernatant was discarded. Crude EPS precipitant was collected and mixed with 5 ml of water. Water and crude EPS mixture was dialyzed using a commercially available dialysis bag (12 to 14 kDa) and DI water at 4 °C for 48 h. The EPS sample inside each bag was collected and dried in a Speed Vacuum Concentrator (ScanSpeed 40, SCANVAC; LaboGene) at 2000 rpm and 35 °C for 24 h. The EPSs in the media supernatants were then quantified by the phenol–sulfuric acid method in a microplate format using glucose as the reference standard [35]. EPS yields ranged from 0.12 ± 0.04 to 59.99 ± 0.91 g/l. The highest EPS-producing LAB strains were selected for further analysis.

### Structural analysis

The monosaccharide composition of EPS was analyzed after hydrolysis. Hydrolysis of EPS (10 mg) was performed with 2 ml of 2 M Trifluoroacetic acid (TFA) in a sealed tube at 110 °C for 6 h, and the TFA was removed by speed vacuum. The dried hydrolyzed samples were dissolved in distilled water (1 ml) and analyzed by HPLC (Thermo Fisher Scientific) [36]. The HPLC system consisted of an Ultimate 3000 pump with an auto sampler and Shodex RI-101 (Shodex, Japan) detector. Sugars were analyzed by sugar-pak (Waters, 300*6.5 mm, USA) column maintained at 70 °C. Ten microliter samples were injected and eluted with triple distilled water at a flow rate of 0.5 ml/min. GPC and NMR analyses of the EPS produced was performed by the National Instrumentation Center for Environmental Management (NICEM) at Seoul National University Molar mass was determined by GPC [Thermo Dionex HPLC, Ultimate 3000 RI System (Thermo Fisher Scientific)]. A calibration curve was established with Pullulan standards of Mw 0.342, 10, 113, 393, 805 kDa dissolved in water containing 0.1 M sodium azide (NaN_3_). Three columns, Waters Ultrahydrogel 120, Waters Ultrahydrogel 500 and Waters Ultrahydrogel 1000, with respective exclusion volumes of 5 × 10^3^ and 1 × 10^6 ^Da, were connected in series. The injected sample volume was 50 μl and the mobile phase was the same sodium azide buffer used to dissolve the sample, flowing at 1 ml/min at a temperature of 40 °C. NMR analysis was conducted using a High Resolution NMR Spectrometer (AVANCE 600, Bruker, Germany). Briefly, *W. confusa* VP30 was cultured in mMRS broth with 10% (w/v) of sucrose for 24 h anaerobically. The collected EPS was purified in the manner previously mentioned. 10 mg samples of purified EPS were dissolved in 1 ml of D2O and the process of ^1^H NMR, ^13^C NMR and 2D NMR was heteronuclear single-quantum coherence (HSQC), and Heteronuclear Multiple Bond Correlation (HMBC), at 600 MHz at 25 °C.

### Safety evaluations of *W. confusa* VP30

A safety assessment of *W. confusa VP30* via ammonia production, hemolysis of blood cells and antimicrobial susceptibility analysis was conducted according to previously reported methods [25]. To evaluate the concentration of secreted ammonia, *W. confusa* VP30 was cultured in Brain Heart infusion (BD Bacto™BHI, LOT 8114937), and the cell-free broth supernatants were recovered after centrifugation at 10,000×*g* at 4 °C for 30 min. The BHI was adjusted to pH 7 using 1 N NaOH before cell inoculation. As previously mentioned, two types of chemical reagents were prepared. The 1st reagent consisted of 1 g phenol and 0.005 g sodium nitroferricyanide dehydrate (Na_2_[Fe(CN)_5_NO]·2H_2_O) dissolved in 100 ml DI water, and the 2nd reagent consisted of 500 mg NaOH and 0.04 g NaClO dissolved in 100 ml DI water. Aliquots (100 μl) of Solutions 1 and 2 were added to Corning^®^ 96 well plates (Costar # 3595, Corning, NY, USA) with 10 μl of the cell-free broth supernatants of *W. confusa* VP30. An ammonia solution (Junsei Chemical, Japan) was used as a control solution. The 96 well plates were incubated in 25 °C for 1 h, and spectroscopy analysis was conducted at 625 nm. Cell-free BHI broth was used as a negative control and the ammonia concentration was assessed using a standard curve. Three replications of this test were conducted for statistical analysis of ammonia production from *W. confusa* VP30.

To evaluate microbial hemolytic properties, *W. confusa* VP30 was anaerobically cultured in BHI agar supplemented with 1.5% agar, 10% of sucrose and 5% horse blood at 37 °C for 2 days. *Listeria ivanovii* subsp. ivanovii ATCC 19119, a positive control for hemolysis, was aerobically cultivated in BHI agar at 37 °C for 2 days. According to our previous protocol [25], the plate was then observed for the presence of microbial hemolysis by holding the plate to a light source and looking through both sides of the plate. Strains showing blood lysis (transparent region) around the colony were classified as microorganisms having hemolytic (β-hemolytic) characteristics. Strains that produced green-hued zones around the colonies (α-hemolysis) or no hemolysis on the blood plates (γ-hemolysis) were considered non-hemolytic.

Minimum inhibitory concentration (MIC) tests were conducted to evaluate antimicrobial susceptibility [25]. Sixteen antimicrobial agents were used: ampicillin sodium salt (Sigma, Lot# BCBW1243), carbenicillin disodium salt (Sigma, Lot# 126M4775V), chloramphenicol (Sigma, Lot# SLBR8869V), clindamycin hydrochloride (Sigma, Lot# 021M1533), dicloxacillin sodium salt hydrate (Sigma, Lot# SZBD263XV), erythromycin (Sigma, Lot# WXBC4044V), gentamicin sulfate (Sigma, Lot# SLBP3082V), kanamycin sulfate (Sigma, Lot# 066M4019V), methicillin sodium salt (Sigma, Lot# BCBR6817V), metronidazole (Sigma, Lot# MKBZ3056V), neomycin sulfate (Sigma, Lot# LRAB3300), phosphomycin disodium salt (Sigma, Lot# 096M4031V), polymyxin B sulfate salt (Sigma, Lot# 126M4071V), spectinomycin sulphate (Sigma,Lot# LRAB3670), streptomycin sulfate salt (Sigma, Lot# SLBT8451), tetracycline (Sigma, Lot# 126M4769V), vancomycin hydrochloride (sigma Lot# LRAB3620). Each of the antibiotic powders were dissolved and diluted in appropriate diluents and filter sterilized prior to addition to LAB susceptibility test medium (LSM) supplemented with cysteine (LSM-Cys) broth medium, composed of 90% of Iso-Sensitest Broth (IST)(Mbcell Lot#P2745063MS, Seoul, Korea) and 10% of MRS(BD Difco™, Franklin Lakes, NJ, USA) broth medium. Serial dilutions of antimicrobial agents ranging from 1024 to 0.0032 μg/ml were prepared. MIC values for all bacterial isolates were determined by the ISO 10932:2010 broth microdilution procedure [37]. The LSM-Cys broth medium supplemented with 0.03% (w/v) l-cysteine HCl containing antibiotics at different concentrations was used to prepare each well of a microwell plate. The inoculum was adjusted to a turbidity equivalent to 0.16 to 0.2 at 625 nm as measured by a spectrophotometer (SpectraMax 190 Microplate reader, Molecular Devices). The solution corresponded to approximately 3 × 10^8^ cfu/ml. Each inoculum was added to a double strength LSM-Cys broth medium at a rate of 0.2%. A 50 μl diluted bacterial suspension was added to each well; no negative control well was employed. The microdilution plates were prepared with a series of twofold dilutions of antibiotics. The microdilution plates were incubated at 37 °C for 48 h in an anaerobic (5% CO_2_, 10% H_2_ and 85% N_2_) chamber. The MIC was defined as the lowest concentration of antibiotic giving a complete inhibition of visible growth in comparison to an antibiotic-free control well. The experiments were replicated three times.

### Statistical analysis

To evaluate EPS production levels, statistical analysis was performed with one-way ANOVA using SPSS 24.0 at a significance level of *p *< 0.05.

## Results and discussion

### Selection of high EPS-producing cell strains

Multiple groups have screened and identified LAB capable of producing EPSs by cell and colony phenotypes in solid medium. This visual cell classification tool has been widely used for many years to identify EPS producing LAB from naturally occurring microbiota as a preselection process. This preselection process has the advantage of being able to screen multiple samples rapidly without special and expensive tools. However, this cell screening process is almost impossible to standardize because colony morphological differences are distinguished via subjective interpretation by the researcher [33]. In addition, some extracellular macromolecules and polymers from LAB might not show significant slime-forming properties and may thus potentially be misidentified as false negatives. According to Rühmann et al. [38], one option to overcome this experimental issue is comparison of colony characteristics (shape, size, pigmentation, etc.) between induced and non-induced EPS production conditions. Since microbial culture conditions and the carbon source of the medium significantly affect EPS productivity and metabolite characteristics [39], effective substrate dependent EPS-producing LAB screening can be achieved by culturing selected cell strains on media supplemented with various carbon sources.

**Fig. 1:**
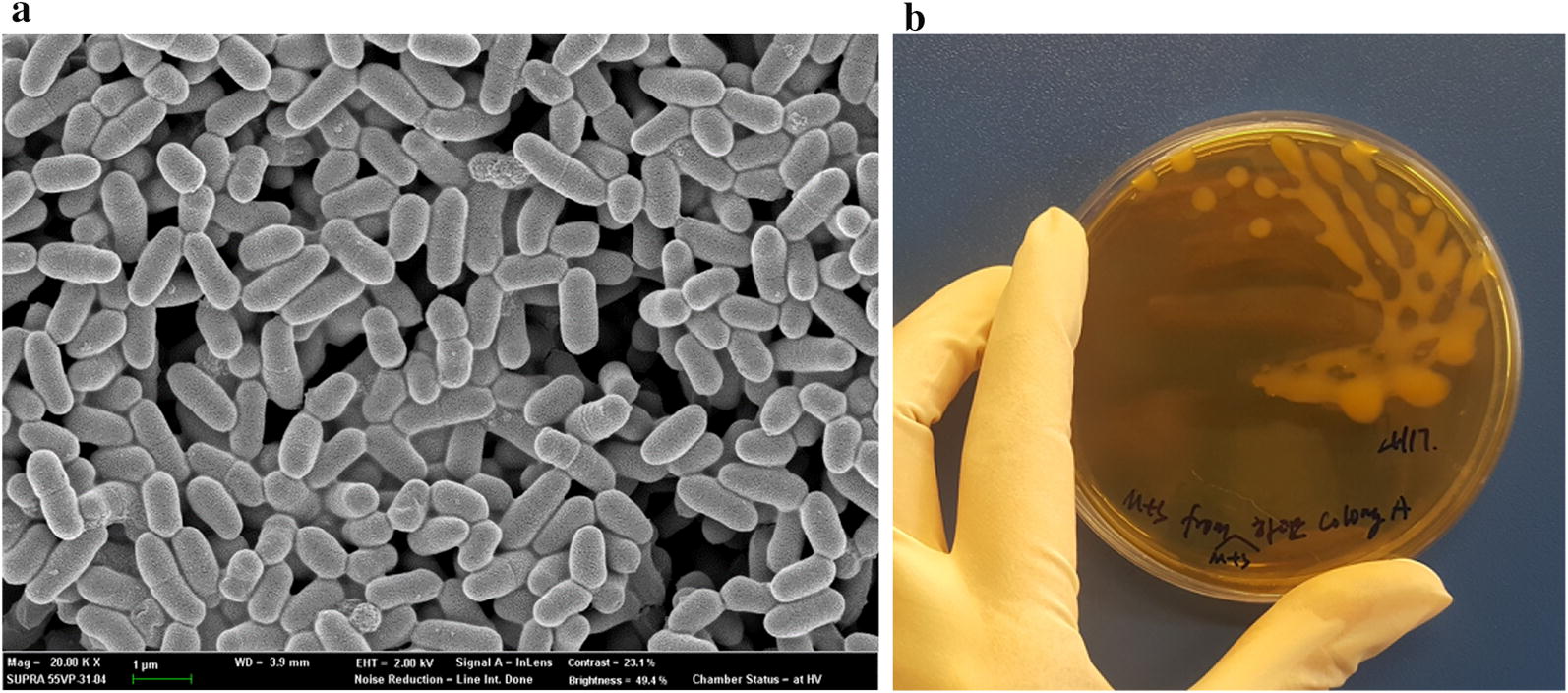
**a** Scanning electron microscope images of *W. confusa* VP30 isolated from a fecal sample from a 5 year old boy, and **b** Morphological aspects of EPS production by *W. confusa* VP30 grown under mMRS with 10% sucrose culture for 24 h

In this work, a total of 156 morphologically different human origin LAB cell colonies were screened from five young (1 to 6 years old) children’s fecal samples using selective media (i.e. LBS or TOS-MUP) as a prescreening step. All 156 LAB colonies were subcultured in commercially available MRS, and pure microbial cultures were obtained by streaking on agar plates. The pure LAB cultures were then cultivated in seven kinds of mMRS media containing one of seven sugars (glucose, sucrose, galactose, lactose, fructose, raffinose and maltose) or commercially available MRS plates. Stickiness was visually and qualitatively observed by touching the microbial colonies with a sterilized inoculation loop or sterilized toothpicks. All 156 of the LAB colonies grown in sucrose showed the most sticky and viscous characteristics versus those grown with other carbohydrate sources. From these, 10 putative EPS-producing LAB candidates were chosen for further study due to their mucoid, slimy, and string-forming appearance. The 16s rRNA of each of these colonies was sequenced and the crude EPS was extracted and obtained by the protocol described by Mendi et al. [35]. Crude EPS solutions from these 10 LAB cultures were used to assess their total carbohydrate content via phenol–sulfuric acid assay. Data comparison with NCBI database sequences for the variable region of the 16S rRNA gene to the species level identified eight out of 10 putative EPS-producing LAB as belonging to the genus *Lactobacillus* and other two to *Weissella* and *Bifidobacterium*. The EPS production of each LAB strain is shown in Table 1. *W. confusa* VP30 (Fig. 1a) produced significantly more EPS than the other LAB strains with significant mucoid appearance (Fig. 1b). In the mMRS broth, the *W. confusa* VP30 produced 59.99 ± 0.91 g/l of EPS, while the other identified LAB strains produced < 0.3 g/l of EPS.

**Table 1:** Overall ranking of isolated strains based on EPS production in mMRS with 10% sucrose (w/v)

No.	16s rRNA	Strain	EPS produced, mean ± SD (g/l)	Host gender	Host age
1	*Weissella confusa*	VP30	59.99 ± 0.91^A^	Male	5
2	*Bifidobacterium longum* subsp. *longum*	JH628	0.29 ± 0.06^B^	Male	1
3	*Lactobacillus plantarum*	JH43A	0.28 ± 0.02^B^	Female	4
4	*Lactobacillus plantarum*	JH102	0.26 ± 0.07^B^	Male	3
5	*Lactobacillus fermentum*	JH43B	0.25 ± 0.11^B^	Female	4
6	*Lactobacillus paracasei*	JH907	0.23 ± 0.02^B^	Female	3
7	*Lactobacillus plantarum* subsp. *plantarum*	JH917	0.21 ± 0.04^B^	Male	1
8	*Lactobacillus plantarum*	JH639	0.21 ± 0.01^B^	Female	4
9	*Lactobacillus rhamnosus*	JH914	0.19 ± 0.04^B^	Male	1
10	*Lactobacillus paracasei*	JH124	0.12 ± 0.04^C^	Female	4

All microorganisms were anaerobically cultured in glucose-free basal MRS supplemented with 10% (w/v) sucrose at 37 °C for 24 h. One-way ANOVA followed by Duncan’s post hoc test statistical analyses were performed. EPS produced (g l^−1^) ± SE with different letters (where A > B > C) are significantly different at *p *< 0.05 (*n *= 3)

*Weissella* was originally classified as *Leuconostoc* or *Lactobacillus*. However, distinctive biochemical features in *Leuconostoc* or *Lactobacillus* were identified by Collins et al. and reclassified as *Weissella* in 1993 [40]. As a naturally occurring microbiota, *Weissella* has been widely engaged in spontaneous lactic acid fermentation and coolship alcoholic beverage fermentations [41], and various *Weissella* strains have recently attracted attention from academia due to their high EPS production ability. Unlike other studies that utilized food-origin bacteria, we isolated and used human-origin strains in this study. The major carbon sources that induce high EPS production varies depending on the microorganism [33]. Sugar transportation pathways, enzyme synthesis metabolism and sugar-nucleotide precursors can be regulated by catabolite repression [33]. In the screening step, all the carbon sources mentioned in the previous study were used. These results show that sucrose is the main carbon source for the production of EPS, which is consistent with previous studies [42, 43]. As shown in Table 2, *Weissella* spp. have higher EPS production capacity than other LAB strains. However, the EPS production properties of *W. confusa* VP30 in this report are significantly higher than those previously reported.

**Table 2:** Various LAB culture conditions utilized for EPS quantitative analysis by other groups

Strain	Source	Medium	Type of EPS	Molecular mass (Da)	Production (g/l)	Method	Standard	References
*W. confusa* VP30	Young children feces	MRS + 10% sucrose	Dextran	3.8 × 10^6^	60.0 ± 0.9	Phenol–sulfuric acid method	Glucose	This work
*W. cibaria* WC4	Sourdough	MRS + 10% sucrose 30 °C 6 days	Glucan	1.1-1.3 × 10^4^	7.9 ± 0.1	Phenol–sulfuric method	–	[46]
*W. cibaria* MG1	Sourdough	MRS + 10% sucrose 30 °C 48 h	Dextran	7.2 × 10^8^	36.4	–	–	[78]
*W. confusa* KR78067*6*	Idli batter	MRS + 2% sucrose 30 °C 48 h	Galactan	ND	17.2	Dry weight	–	[88]
*W. confusa* NH02	Nham	MRS + 4% sucrose 37 °C 12 h	ND	1.13 × 10^6^	18.08	–	–	[48]
*W. hellenica* SKkimchi3	Kimchi	MRS + sucrose 20 °C 48 h	Glucan	2.03 × 10^5^	5.12	–	–	[49]
*W.* c*ibaria* 27	Kimchi	60 g/l of sucrose 22 °C and pH 6.2 for 24 h	Glucan	1.2 × 10^7^	24.8	Lyophilized dry weight		[47]
*Leu. pseudomesenteroides*	Soybean paste	5% sucrose 30 °C for 48 h	Glucan	–	12.5	Phenol sulfuric acid method [89]		[60]
*W. confusa*	–	80 g/l of sucrose	–	–	25.2	Freeze drying dry weight		[90]
*Leu. citreum* B-2	Homemade fermentation product of pineapple	75 g/l sucrose	Glucan	3.77 × 10^6^	28.3	Lyophilized dry weight		[51]
*W. confusa* OF126	Ogi	24.0 g/l sucrose, pH (7.00)	Glucan	1.1 × 10^6^	3.10	Lyophilized dry weight		[50]
*Leu. Mesenteroides* 109	Plant	20 g/l of fructose, 50 g/l of sucrose	Glucan	1.4 × 10^6^	19.0	Polymer dry mass 42 °C 48 h		[52]
*W. cibaria*	Fermented sauropus androgynus	Sucrose	Glucan	–	6.4	Lyophilized dry weight		[45]
*W.* sp. PSMS4-4	Plasom	5% White-sugar 30 °C	Glucan	–	8.7	Phenol sulfuric acid method [89]	Glucose	[91]

### Evaluation of EPS constituents

**Fig. 2:**
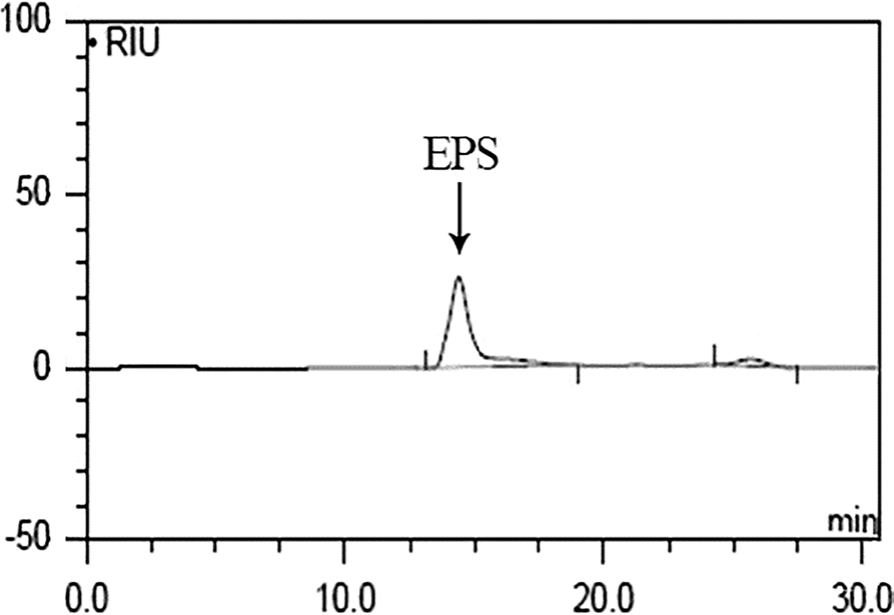
Elution profile and determination of molecular weight of *W. confusa* VP30 EPS by gel filtration on Thermo Dionex HPLC with Ultimate 3000 RI System. Pullulan of multiple molecular mass were used as the standard

The carbohydrate composition of EPS isolated from *W. confusa* VP30 was consisted predominantly of glucose. The weight-average molecular weight (Mw) of the EPS was determined by GPC analysis with three columns (i.e. Waters Ultrahydrogel 120, Waters Ultrahydrogel 500 and Waters Ultrahydrogel 1000), where a typical sharp symmetrical peak was observed (Fig. 2) [44, 45]. *W. confusa* VP30 produced 3.8 × 10^6^ Da EPS which is significantly larger than those from other *Weissella* spp. (Table 2) [46–52].

**Fig. 3:**
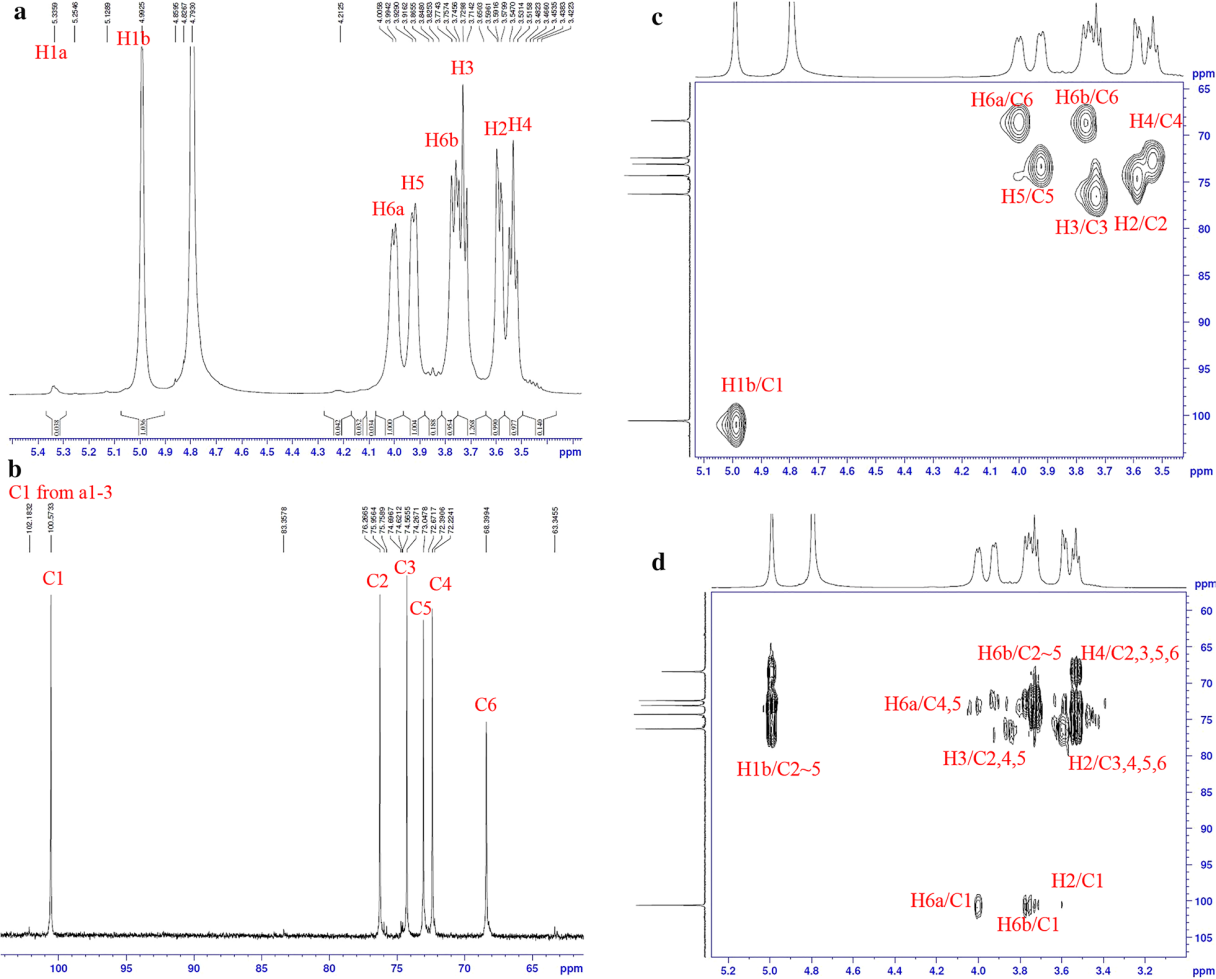
1D NMR spectrum of purified EPS from *W. confusa* VP30 in D2O: **a** ^1^H NMR (600 MHz); **b** ^13^C NMR (150 MHz). **c** HSQC spectrum; **d** HMBC spectrum

The ^1^H NMR spectrum of the *W. confusa* VP30 produced EPS is shown in Fig. 3a. The peaks were distributed into two regions, upfield of 3.5–4.0 ppm and downfield of 4.9–5.3 ppm. This was in agreement with the 1H NMR spectrum of dextran where the proton signals at C-2, C-3, C-4, C-5, and C-6 were found in the range of 3–4 ppm while C-1 was found in the 4–6 ppm region [47, 53–55]. The chemical structure of EPS should be alpha as the chemical shift is 100.57/4.99 ppm for ^13^C/^1^H while 5.3–5.7 ppm for ^1^H and 103–106 ppm for ^13^C belongs to beta form [47]. From the spectrum, two peaks of anomeric protons were observed. The high intensity peak at 4.99 ppm was referred to as the anomeric proton of the *α* (1 → 6) linkages in the main chain [47, 56], whereas the low intensity peak at 5.33 ppm represented the anomeric proton of the *α* (1 → 3) branch linkages [56]. A ratio of 96.5:3.5 was obtained from the integration of the relative intensity of the signal at 4.99 and 5.33 ppm. This result suggests that the dextran was composed of 96.5% *α* (1 → 6) linkages in the main chain and 2.6% *α* (1 → 3) branch linkages. The ^13^C NMR spectrum revealed six peaks appearing between 68 and 100 ppm (Fig. 3b). In previous studies, anomeric carbon signals were generally found downfield, in the region of 95–105 ppm. The signals in 95–101 ppm were related to ɑ-anomeric carbons, and the signals in 101–105 ppm were due to β-anomeric carbons in ^13^C NMR spectra, which corresponded with the ^1^H NMR spectra [57]. Carbon signals at C-2, C-3, C-4, and C-5 were found in the range of 70–75 ppm, while C-6 was found in the upfield region at about 60 ppm [58]. Moreover, dextran branch linkage signals were found around 77–85 ppm [59]. In this study, the signal at 100.57 ppm was clearly due to the anomeric carbon C-1, while the signal at 68.39 ppm was due to C-6. This peak was also observed at a lower intensity compared to the other peaks since some parts of the *α* (1 → 6) linkages had been replaced by *α* (1 → 3) branches. However, the signal referring to branch linkages around 77–85 ppm was absent due to the small amount of *α* (1 → 3) linkages. To assign the signals at 72.39, 73.04, 74.26, and 76.26 ppm, the data from 2D NMR were also analyzed. HSQC(c) and HMBC(d), which provided the correlation between ^1^H and 13C direct bond and two bonds, respectively, were used to confirm the structure of the polysaccharide (Fig. 3c, d). From the HSQC spectrum, seven protons in the glucose residue were found to correlate. The correlation of 1H at 4.99 ppm and ^13^C at 100.57 ppm confirmed the presence of *α* (1 → 6) glycosidic bond. Other assignments from the HSQC and HMBC analysis of each position are presented in Table 3. The NMR results are similar to dextran produced by *W. confusa* R003 [56] and this HSQC, and HMBC similarities confirmed that the dextran was composed of glucose units linked with 96.5% *α* (1 → 6) glycosidic bonds and 3.5% *α* (1 → 3) branches. This data is consistent with others’ data and reconfirms the special chemical structure of *Weissella* EPS (dextran) which shows high linearity with long chains and low branching [56, 60]. The highly linear dextran from *W. confusa* VP30 could be used as a food modifier for thickening, viscosifying and also as a potential soluble fiber which can act as a prebiotic [61].

**Table 3:** Assignment of ^1^H/^13^C chemical shift of dextran from *W. confusa* VP30 correlated with dextran from Netsopa et al. [56]

Atom position	1	2	3	4	5	6
*W. confusa* R003 [56]						
^1^H	4.98	3.58	3.72	3.52	3.9	3.76/3.98
^13^C	100.28	74.1	76.09	72.28	72.88	68.33
*W. confusa* VP30						
^1^H	4.99	3.59	3.72	3.54	3.91	3.77/3.99
^13^C	100.57	74.26	76.26	72.39	73.04	68.39

### Safety evaluation

Multiple *Weissella* strains have been detected and screened from traditional fermented foods as naturally occurring microbiota. Specifically, *W. viridescens*, *W. confusa* and *W. cibaria* were extracted from fermented vegetables [62–64] (e.g. kimchi and sauerkraut); from fermented meat products [65, 66] (i.e. Italian fermented sausages and Greek dry salami), *W. hellinica*, *W. paramesenteroides*, *W. viridescens*, *W. paramesenteroides*, *W. minor* and *W. halotolerans* were isolated. *W. hellenica, W. halotolerans* and *W. viridescens* were identified from fermented dairy product [67–69] (e.g. Romanian, mozzarella and cheddar cheeses).

Many research groups are trying to identify the biofunctional activities of *Weissella* spp. and practically apply them to food, feed, and/or cosmetic products as a starter cultures and/or additives [70–73]. Recently, Lee et al. [74] proposed two *W. confusa* spp. (i.e. *W. confusa* 20 and 31) screened from human fecal samples as potential probiotic bacteria due to their high antibiotic sensitivity and cell adhesion properties. *W. kimchii* PL9023 was isolated from the vagina of a healthy premenopausal woman and has been proposed as a potential probiotic bacteria due to its inhibitory effects on fungal and bacterial vagina infection by *Candida albicans*, *Escherichia coli*, *Staphylococcus aureus* and *Streptococcus agalactiae* [75]. Fusco et al. [76] reported six types of bacteriocin-producing *Weissella* spp. (i.e. *W. paramesenteroides*, *W. hellenica*, and *W. cibarian)*. Recently, Kang et al. [77] practically applied *W. cibaria* to chewing gum and evaluated cell survival rates with changed environmental factors (e.g. temperature and additives). Zannini et al. [78] applied *W. cibaria* MG1 isolated from cereal to wort (beer substrate) production. Recently Abriouel et al. [79] published reviews of anti-viral, anti-tumoral, anti-obesity, anti-inflammatory, and antioxidant activities of *Weissella* and suggested the possibility of their use as a starter culture.

Although the superior functionality and universality of the *Weissella* spp. in fermented foods is of interest within academia, several studies have also warned of the possibility for side effects (e.g. hemolytic activity, production of toxic substances during ammonia metabolism, and antibiotic resistance by their host). Recently, Abriouel et al. [79] conducted in silico gene expression analysis with several *Weissella* spp. and reported the presence of antibiotics (e.g. daunorubicin, fosfomycin, methicillin and tetracycline) resistance, hemolysin gene in these microorganisms. Jung and Lee [80] evaluated the antibiotic resistance, hemolysis and biogenic amine production properties of 45 *Weissella* strains (i.e. *W. cibaria* [n = 33], *W. confusa* [n = 11] and *W. paramesenteroides* [n = 1]) isolated from kimchi for kimchi starter development. They reported that all *Weissella* strains (n = 45) showed significant antibiotic resistance against streptomycin when the disk diffusion test was applied. Specifically, 54.5% of tested *W. cibaria* (n = 18) was resistant to gentamicin, and 15.2% to penicillin G (n = 5). 8.5% of *W. confusa* was resistant to gentamicin (n = 3). In their study, only one species of *W. paramesenteroides* (n = 1) was used, and this strain showed antibiotic resistance against gentamicin. In addition, some LAB are known to produce ammonia, which is generally regarded as a toxic substance produced by edible microorganisms via utilization of nitrogen sources during fermentation [81, 82]. Therefore, safety evaluation is critically required for further industrial applications even though *W. confusa* VP30 has been isolated from the feces of healthy children.

**Fig. 4:**
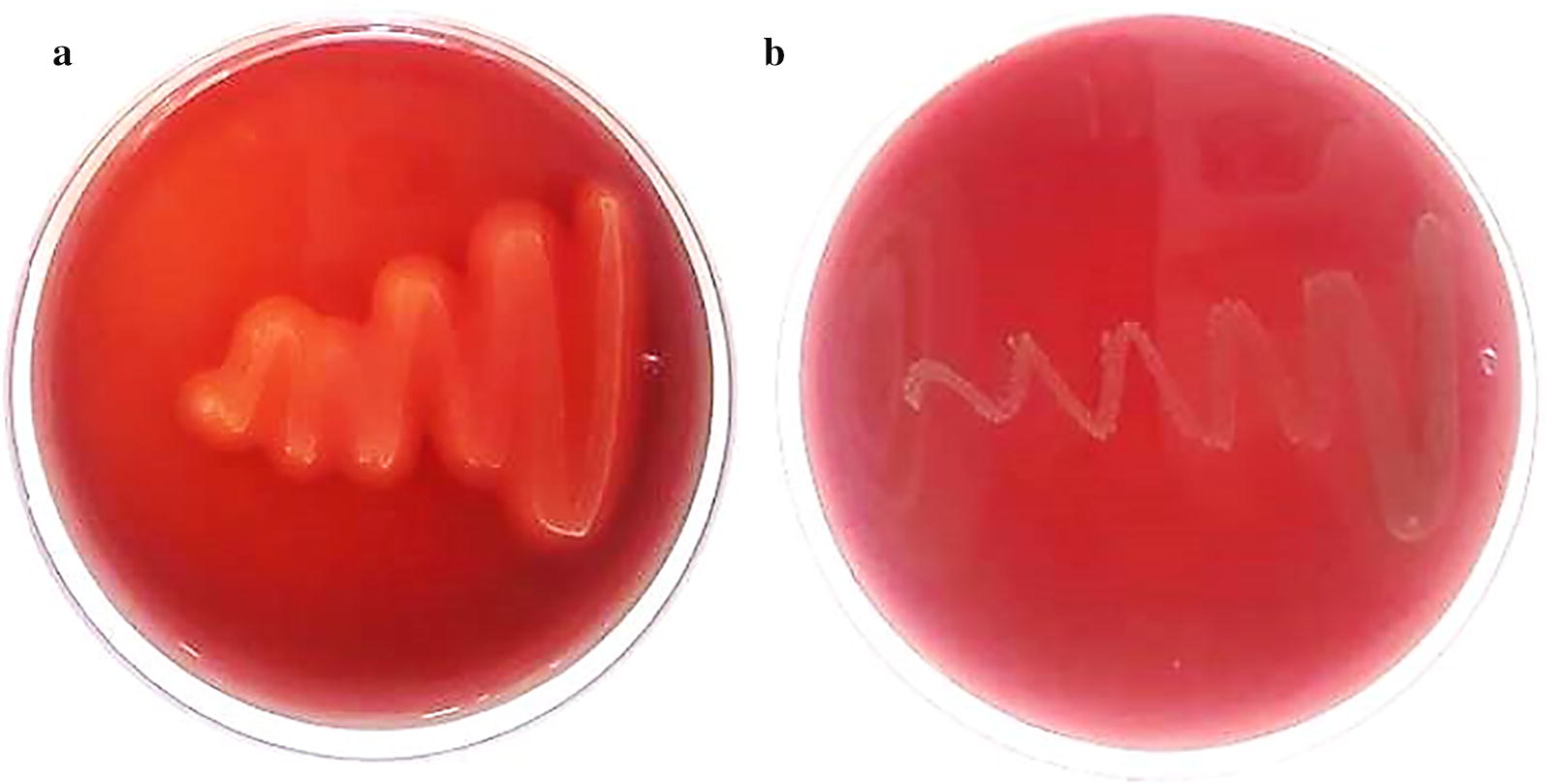
**a** Comprehensive lysis of red blood cells was observed with clear zones around the *Listeria ivanovii* subsp. *ivanovii* ATCC 19119 (positive control). **b** *W. confusa* VP30 growth with no blood cell lysis on the blood agar. The hemolytic activity of each strain was assessed in triplicate

*Listeria ivanovii* subsp. *ivanovii* ATCC 19119 and *W. confusa* VP30 were streaked on BHI with 1.5% agar and 5% horse blood and incubated at 37 °C for 48 h. The microorganisms that produced clear and/or transparent zones around the cell colonies were classified as β-hemolytic. Microorganisms that did not generate any visual changes on the blood agar were regarded non hemolytic cell. Figure 4 shows significant transparent and clean zones around the β-hemolytic positive control *Listeria ivanovii* subsp. *ivanovii* ATCC 19119, due to the complete lysis of red blood cells on the media. Other strains belonging to the *Weissella confusa* species have been reported as alpha- hemolytic [83]. However, *W. confusa* VP30 generated no clear or transparent zones on the blood agar around their microbial colonies, indicating that *W. confusa* VP30 is non-hemolytic (a.k.a. γ-hemolytic).

LAB’s ammonia production property can potentially be related to host gastrointestinal health. Some researchers have reported ammonia production properties of *Lactobacillus* spp. [82, 84]. Therefore it is necessary to evaluate *W. confusa* VP30’s ammonia production capability and demonstrate its safety for further application in processed food production [25]. A colorimetric assay was used to assess the level of ammonia in the media after incubation. *Enterobacter cloacae* KCTC 2361, the positive control for ammonia production, produced 11.5 ± 0.9 µg/ml of ammonia while *W. confusa* VP30 was negative.

Excessive use of antibiotics has been reported to contribute to increasing numbers of pathogens having antibiotic resistance, which is considered a serious threat to the treatment of bacterial infection. Although many LAB are not commonly regarded as pathogenic bacteria, it is desirable that they be sensitive to commercially available antibiotics at low concentrations. To evaluate the antimicrobial resistance properties of *W. confusa* VP30, we conducted MIC tests based on the International Organization for Standardization (ISO) standard protocol. Based on the MIC criteria for microorganisms used in food or feeds prescribed in EFSA [85], *W. confusa* VP30 was found to have no significant resistance to gentamicin, kanamycin, streptomycin, tetracycline, clindamycin, ampicillin (< 4 µg/ml, Table 4). Although there are no reference concentration and/or guidelines for methicillin, dicloxacillin hydrate, polymyxin B sulfate, bacitracin, neomycin, carbenicillin and spectinomycin resistance, *W. confusa* VP30 was found to be sensitive to these antibiotics as well, with MIC values ranging from 1 to 32 μg/ml. On the other hand, *W. confusa* VP30 was resistant to erythromycin and chloramphenicol. (MIC ranging from 2 to 8 μg/ml). Compared with other *W. confusa* strains [86, 87], *Lactobacillus plantarum* ATCC 14917 [37] and *Lactobacillus paracasei* ATCC334 [37], *W. confusa* VP30 have similar or lower MIC concentrations of gentamicin, kanamycin, tetracycline, clindamycin, ampicillin and erythromycin.

**Table 4:** MIC distributions of seventeen antibiotics for *W. confusa* VP30 isolates from a child fecal sample determined by the ISO 10932:2010 broth microdilution procedure

	*Weissella confusa* VP30 (μg/ml)	*Weissella confusa* [87] (μg/ml)	*Weissella confusa* [86] (μg/ml)	*Lactobacillus paracasei* ATCC334 [37] (μg/ml)	*Lactobacillus plantarum* ATCC14917 [37] (μg/ml)	EFSA cut-off of *Leuconostoc* [85] (μg/ml)
Gentamicin	1	≤ 2	3	1 to 4	–	16
Kanamycin	4	–	–	16 to 64	–	16
Vancomycin	> 128	Resistant	> 256	–	–	nr
Streptomycin	32	–	–	8 to 12	–	64
Tetracycline	2	4 to 8	–	1 to 4	8 to 32	8
Clindamycin	< 0.032	≤ 0.5	0.06	0.06 to 0.25	0.5 to 4	1
Ampicillin	0.5	0.5	0.5	0.5 to 2	0.25 to 2	2
Erythromycin	2	≤ 0.25	0.13	0.06 to 0.5	0.25 to 2	1
Methicillin	32	–	–	–	–	–
Dicloxacillin salt hydrate	8	–	–	–	–	–
Polymycin B sulfate salt	32	–	–	–	–	–
Bacitracin	1	–	–	–	–	–
Methronidazole	512		> 256	–	–	–
Phosphomycin disodium salt	> 1024		–	–	–	–
Neomycin	2	–	–	–	–	–
Carbenicillin	2	–	–	–	–	–
Chloramphenicol	8	8	–	2 to 8	4 to 16	4
Spectinomycin	32	–	–	–	–	–

## Conclusion

The objective of this work was to screen and characterize high level EPS-producing LAB from healthy children’s feces for potential functional food applications. As a prescreening process, 156 LAB cell strains were screened and isolated using LAB selective media (i.e. LBS and TOS-MUP) and cultivated in eight different modified MRS broths containing various sugars. When the 156 LAB strains were cultivated in media supplemented with sucrose as the major carbon source, the viscosity of microbial colonies was increased. The 10 most mucoid and slimy colony-producing colonies were selected based on their solid agar media phenotypes as EPS positive strains. 16S rRNA gene sequencing analysis showed that these high EPS-producing cell strains were members of *Weissella confusa*, *Bifidobacterium longum*, *Lactobacillus plantarum*, *Lactobacillus fermentum*, *Lactobacillus paracasei* and *Lactobacillus rhamnosus* genus and species. Among them, *W. confusa* VP30 showed significantly higher EPS productivity compared to the other cell strains and was selected for further testing. The maximum EPS production by *W. confusa* VP30 was 59.99 ± 0.91 g/l. Structural analysis of the released EPS fraction by NMR revealed that *W. confusa* VP30 can produce dextran of 3.8 × 10^6^ Da, containing glucose units linked with 96.5% *α* (1 → 6) glycosidic bonds and 3.5% *α* (1 → 3) branches. Safety assessment of *W. confusa* VP30 was accomplished via ammonia production, hemolysis of blood cells and antibiotic resistance analyses. There is no evidence that this LAB strain is unsafe for human consumption, based on our results. Considering the active EPS producing capability and safety of *W. confusa* VP30, this microbial cell strain can be considered for use by the food industry.

## Data Availability

The datasets used and/or analysed during the current study are available from the corresponding author on reasonable request.
